# Mebeverine for Pediatric Functional Abdominal Pain: A Randomized, Placebo-Controlled Trial

**DOI:** 10.1155/2014/191026

**Published:** 2014-06-25

**Authors:** Zahra Pourmoghaddas, Hossein Saneian, Hamidreza Roohafza, Ali Gholamrezaei

**Affiliations:** ^1^Child Growth and Development Research Center, Isfahan University of Medical Sciences, Hezar Jerib Avenue, Isfahan 81746-75731, Iran; ^2^Psychosomatic Research Center, Isfahan Cardiovascular Research Institute, Isfahan University of Medical Sciences, Isfahan 81746-75731, Iran; ^3^Poursina Hakim Research Institute, Isfahan 81465-1798, Iran

## Abstract

We evaluated the effectiveness of an antispasmodic, mebeverine, in the treatment of childhood functional abdominal pain (FAP). Children with FAP (*n* = 115, aged 6–18 years) received mebeverine (135 mg, twice daily) or placebo for 4 weeks. Response was defined as ≥2 point reduction in the 6-point pain scale or “no pain.” Physician-rated global severity was also evaluated. Patients were followed up for 12 weeks. Eighty-seven patients completed the trial (44 with mebeverine). Per-protocol and intention-to-treat (ITT) analyses were conducted. Treatment response rate in the mebeverine and placebo groups based on per-protocol [ITT] analysis was 54.5% [40.6%] and 39.5% [30.3%] at week 4 (*P* = 0.117 [0.469]) and 72.7% [54.2%] and 53.4% [41.0] at week 12, respectively (*P* = 0.0503 [0.416]). There was no significant difference between the two groups in change of the physician-rated global severity score after 4 weeks (*P* = 0.723) or after 12 weeks (*P* = 0.870) in per-protocol analysis; the same results were obtained in ITT analysis. Mebeverine seems to be effective in the treatment of childhood FAP, but our study was not able to show its statistically significant effect over placebo. Further trials with larger sample of patients are warranted.

## 1. Introduction

Chronic abdominal pain is one of the most common complaints of children in pediatricians' offices and can result in distress and discomfort in both the child and the parents [1]. In most cases, the cause of chronic abdominal pain is nonorganic leading to a diagnosis of functional gastrointestinal disorder (FGID), with functional abdominal pain (FAP) being a common diagnosis [2]. The prevalence of FAP is reported variously from 0.3 to 19% (median 8.4%) of children in Western counties [3]. Children with FAP as well as their parents have obviously diminished quality of life [4, 5]. School-aged children with abdominal pain miss their school days by the average of 2.3 days, and 10% of the parents miss their day works [6]. Accordingly, childhood FAP affects the economy and health of the society.

Alternation of gastrointestinal motility [7], visceral hypersensitivity [8], and psychosocial factors [9] are proposed in the pathophysiology of the FGIDs. Since the pathophysiology of FGIDs is not completely understood, treatment of FAP in children remains a challenge for clinicians. Various pharmacological and nonpharmacological therapies are studied up to now, but most of them failed to provide substantial therapeutic effects [10]. It is presumed that in FGIDs a dysregulation within enteric and the central nervous systems results in alternations in sensation and motility and probably causes intolerance to gastric distinction [7, 8, 11]. Along with this presumption, antispasmodics which modulate the smooth-muscle contraction have been investigated as treatments for FGIDs [12]. Mebeverine is a smooth-muscle relaxant with anticholinergic activity. Recent meta-analyses showed that antispasmodics, including mebeverine, are superior to placebo in the treatment of adults with IBS [12–14], though controversies exist in this regard [15].

Few studies have evaluated the effectiveness of antispasmodics in the treatment of childhood FGIDs. One study has shown beneficial effects of peppermint oil for childhood IBS [16], but no randomized trial is available on mebeverine or other antispasmodics in children with FGIDs. Mebeverine is shown to be well tolerated and have no significant adverse event in adult patients [15]. According to the lack of evidence in pediatric patients, we conducted a randomized, placebo-controlled trial on the efficacy of mebeverine in the treatment of FAP in children.

## 2. Materials and Methods

### 2.1. Study Participants

A clinical trial was conducted from February through December 2013 at an outpatient clinic of pediatric gastroenterology in Isfahan City, Iran. Eligible participants were children in the age range of 6 to 18 years who fulfilled the Rome III diagnostic criteria for FAP. The criteria include episodic/continuous abdominal pain at least once per week for at least two months [2]. Children with alarm signs (e.g., anemia, rectal bleeding, etc.) were further evaluated for organic diseases. Those with organic diseases as the cause of abdominal pain and other concomitant gastrointestinal disorders and those with history of receiving antibiotics, antidepressant agents, or probiotics in the preceding two months were not included into the study. The study was approved by the Ethics Committee of the Isfahan University of Medical Sciences and informed consent was obtained from the parents.

### 2.2. Study Design and Sample Size

The study was designed as a randomized, double-blind, placebo-controlled trial. Mebeverine and placebo containing drug bottles were coded by a pharmacist using random numbers in four blocks (generated by software). Allocation was concealed and the attending physician, participants, and outcome assessor were unaware of the drug codes. Based on available evidence on antispasmodics applied for children, we estimated a treatment response of 70% for mebeverine and 40% for placebo [16]. At a power of 80% and a significance level of 0.05, we needed 41 children per group. The trial was registered in Australian New Zealand Clinical Trial Registry (ACTRN12613000158763).

### 2.3. Intervention

The treatment group received mebeverine tablets 135 mg twice daily (Fanak Chemistry Pars Tehran Co., Tehran, Iran) for a duration of 4 weeks. The placebo group received placebo tablets (similar in shape, color, and size) in a same order. Adherence was examined after two weeks of medication by telephone interview and also at the 4-week visit.

### 2.4. Outcome Measures and Follow-Up

The primary outcome measure was treatment response defined as at least 2-point reduction in the Wong-Baker FACES Pain Rating Scale or “no pain” after medication. This pain rating scale is a well-known instrument for measuring pain intensity in children by self-report. Consisting of six faces that show pain effect, the scale ranges from a relaxed face on the left (no hurt scored 0) to a face showing intense pain on the right (hurts worse scored 5). The child was asked to choose the face he/she has at the time of pain [17].

Secondary outcomes during the 4-week medication included the physician-rated global severity and improvement using the Clinical Global Impression Severity and Improvement Scales (CGI-S, CGI-I). The CGI-S and CGI-I are brief 7-point physician-rated scales of the global severity of the illness and improvement by the treatment, respectively. The severity is scored from 1 (normal) to 7 (among the most extremely ill patients) and the improvement is scored from 1 (very much improved) to 7 (very much worse) [18].

Adverse events were assessed after two weeks of medication by telephone interview and also at the 4-week visit using a checklist including common side effects of mebeverine. In case of severe side effects, drug was discontinued. To test durability of the response to medication, primary and secondary outcomes' measurements were repeated 8 weeks after medication period (the 12-week follow-up visit).

### 2.5. Statistical Analysis

Data are presented as mean ± SD or number (percent). Data were assessed for a normal distribution before analyses. Between-group comparisons were done with independent *t*-test and Chi-square test. Equivalent nonparametric tests were applied when appropriate. We compared the two groups regarding the study outcomes based on the per-protocol as well as intention-to-treat (ITT) principles. The last observation carried forward method was applied and participants who did not attend the posttreatment or follow-up visits were considered not to have had any change in scores from the previous visit. The Statistical Package for Social Sciences software version 16.0 (SPSS Inc., Chicago, IL, USA) was used. A two-sided *P* value of less than 0.05 was considered statistically significance in all analyses.

## 3. Results

**Figure 1:**
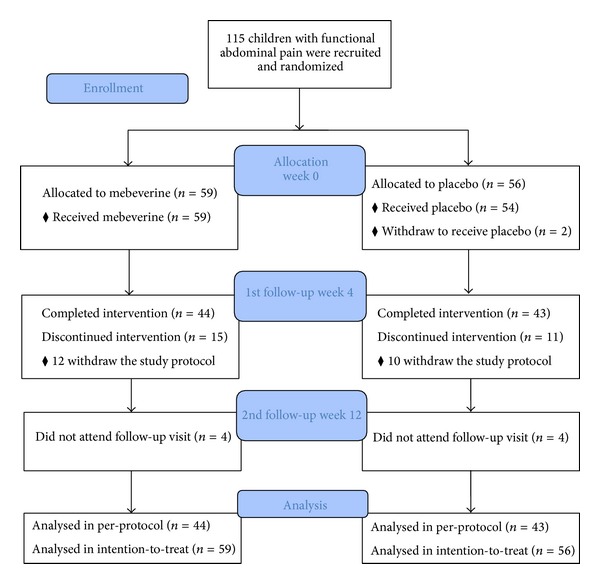
Patients' flow diagram.

During the study period, 115 children with FAP were assigned to either the mebeverine (*n* = 59) or the placebo (*n* = 56) groups. Twenty-four patients withdraw to follow the study protocol, not related to side effects. Three patients from the mebeverine group discontinued medication due to side effects; two had drowsiness and nervousness, and one had nausea. In the placebo group, one patient used antibiotics during the medication period. A total of 87 patients completed the 4-week medication period. Eight patients (4 in the mebeverine and 4 in the placebo group) did not attend the follow-up visit at week 12 (Figure 1, patients' flow diagram). There was no difference between patients who did not follow the study protocol and those who remained in the study regarding demographic factors or baseline values of the study outcome variables.

### 3.1. Baseline Characteristics of the Patients

Demographic data and baseline characteristics are presented in Table 1. Mean age of the total participants was 8.5 ± 2.1 years and 48 (55.1%) were female. There was no significant difference between the two groups in demographic data or baseline characteristics.

### 3.2. Primary and Secondary Outcome Measures

Univariate comparisons of the primary and secondary outcomes between the two groups based on per-protocol and ITT analyses are presented in Table 2. The two groups were not significantly different in change of pain score at 4 weeks (*P* = 0.285) or at 12 weeks (*P* = 0.151) based on per-protocol analysis. The ITT analysis revealed the same result. Treatment response rate in the mebeverine and placebo groups based on per-protocol [and ITT analysis] was 54.5% [40.6%] and 39.5% [30.3%] at week 4 (*P* = 0.117 [0.469]) and 72.7% [54.2%] and 53.4% [41.0] at week 12, respectively (*P* = 0.0503 [0.416]). There was no significant difference between the two groups in change of the CGI-S score after 4 weeks (*P* = 0.723) or after 12 weeks (*P* = 0.870) in per-protocol analysis; the same results were obtained in ITT analysis. The CGI-I score was nonsignificantly lower (indicating more improvement) in the mebeverine group than the placebo group at week 4 (*P* = 0.057), but not at week 12 (*P* = 0.183) in per-protocol analysis. Such difference was not observed in ITT analysis (Table 2).

### 3.3. Treatment Adherence and Side Effects

Patients in the mebeverine and placebo group consumed 81.5 ± 18.7% and 89.4 ± 10.6% of the drugs, respectively. Comparison of side effects between the two groups is summarized in Table 3. The mebeverine group experienced more dry mouth than the placebo group during medication (43.1% versus 23.2%, *P* = 0.047). Other possible side effects were comparable between the two groups.

## 4. Discussion

Antispasmodic agents have been investigated for the treatment of pain-related FGIDs based on the assumption that they reduce smooth muscle spasms in the gastrointestinal tract and therefore can decrease symptoms such as pain. Antimuscarinic/anticholinergic drugs, smooth-muscle relaxants, and selective calcium channel blockers are subtypes of antispasmodic agents [13]. A meta-analysis by Ford and colleagues on 22 studies that evaluated the effectiveness of 12 different antispasmodics in the treatment of IBS showed that otilonium, cimetropium, hyoscine, and pinaverium reduce IBS symptoms. Among these agents, the most qualified evidence was available for hyoscine [14]. However, this meta-analysis was limited regarding data on other antispasmodic agents such as mebeverine [14]. A recent meta-analysis by Martínez-Vázquez and colleagues on 27 trials of antispasmodic agents, including mebeverine, showed that these agents are effective in the treatment of IBS [12]. Another meta-analysis by Poynard et al. also showed the same results [13]. However, the meta-analysis by Darvish-Damavandi and colleagues which only included trials of mebeverine for IBS patients found no statistically significant effects for mebeverine on clinical improvement or relief of abdominal pain [15]. All of these meta-analyses have indicated the very few side effects of antispasmodic agents. The controversial results are related to significant heterogeneity between the included trials.

Despite the available evidence on the efficacy and safety of antispasmodic agents in adult patients with FGID, there is a lack of randomized studies in pediatric patients. We evaluated the effectiveness of mebeverine in the treatment of FAP in children. Based on the per-protocol analysis, we found a relatively higher treatment response rate with mebeverine compared with placebo after 4 weeks of medication and also after 8-week follow-up, but differences were not statistically significant. Such differences were not evident in the ITT analysis. These results could be related to high placebo response and limited sample size of our study as well as high dropout rate. It must be noted that most of the withdrawals to follow the study protocol were not related to drug side effects. Also, it must be noted that while the per-protocol analysis might overestimate the effectiveness of the drug, the ITT analysis might underestimate that. Accordingly, further trials with larger sample of patients are required before a clear conclusion could be made.

There is only one other report available regarding the efficacy of antispasmodic agents in pediatric FGID patients. Kline and colleagues evaluated the effectiveness of peppermint oil in 42 children with IBS. After 2-week treatment, authors found 76% response with peppermint oil compared with 19% with placebo with no adverse events [16]. Several clinical trials up to now have shown the efficacy and safety of peppermint oil in the treatment of IBS in adult patients [19]. Peppermint oil has been superior to placebo for global improvement of IBS symptoms and improvement in abdominal pain with mild and transient side effects [19]. The primary active ingredient of peppermint oil, the menthol, acts as a calcium channel blocker in the intestinal smooth muscles and reduces colonic spasms and associated pain [20]. Peppermint has also analgesic and immune-modulating actions which may contribute to its effects on FGIDs symptoms [21]. However, it must be noted that there are differences in the pathophysiologic mechanisms behind FAP and IBS, and response to antispasmodics may be age related as well. Hence, further trials are required on the efficacy and safety of antispasmodics including peppermint in children with FAP.

In our study, medication duration was 4 weeks and no deterioration of abdominal pain was observed up to 8 weeks after drug discontinuation. Previous trials on mebeverine in adult IBS patients had medication duration of between 4 and 16 weeks, but no specific follow-up period has been reported [15]. As recommended by the Rome Committee, at least 6-month follow-up is required to establish long-term efficacy of the treatment for FGIDs [22]. While there was no report on the effectiveness of mebeverine in childhood FGIDs, it was reasonable to follow up patients for a shorter duration in our study. However, according to the results of this study, further trials with longer duration of medication and follow-up are warranted.

Antispasmodics are generally safe medications. In the meta-analysis by Ford et al., about 14% of adult patients assigned to antispasmodics experienced adverse events compared with 9% allocated to placebo with common side effects included as dry mouth, dizziness, and blurred vision. None of the trials have reported any serious adverse events [14]. The other meta-analysis on trials of mebeverine for IBS also showed that this agent is well tolerated with no significant adverse effects [15]. The results of the present study regarding side effects of mebeverine in children are comparable to studies that evaluated this medication as well as other antispasmodics in adults [14, 15]. We found that mebeverine is generally safe in children with no significant adverse effect. Only 5.3% of the patients had no tolerance to the drug and dry mouth was the most common side effects.

## 5. Conclusions

Mebeverine seems to be effective in the treatment of childhood FAP, though our study was not able to show its statistically significant effect over placebo due to the high placebo response and limited sample of the study. It is, however, safe in children with no significant adverse events and further trials with larger sample of patients are recommended in this regard.

## Figures and Tables

**Table 1:** Baseline characteristics of the patients.

	Mebeverine *n* = 44	Placebo *n* = 43	*P* value
Age, year	8.5 ± 2.0	8.5 ± 2.2	0.839∗
Girl/boy	24 (54.5)/20 (45.4)	24 (55.8)/19 (44.1)	0.538∗∗
Father education			
0–5 y	3 (6.8)	7 (16.2)	0.660∗∗∗
6–12 y	29 (65.9)	24 (55.8)	
>12 y	11 (25)	12 (27.9)	
Family income^†^			
Low income	7 (15.9)	9 (20.9)	0.492∗∗∗
Middle income	25 (56.8)	26 (60.4)	
High income	10 (22.7)	8 (18.6)	
Pain score	3.5 ± 1.0	3.6 ± 0.8	0.557∗∗∗
CGI-S score	4.9 ± 1.1	5.4 ± 0.9	0.056∗∗∗

Data are presented as mean ± SD or number (%). CGI-S: Clinical Global Impression Severity. ∗Independent sample *t*-test, ∗∗Chi-square test, and ∗∗∗Mann-Whitney *U* test. ^†^Based on the Iranian Rial currency. Two patients did not provide information on family income.

**Table 2:** Changes in primary and secondary outcome measures.

	Mebeverine *n* = 44 [59]	Placebo *n* = 43 [56]	*P* value
Change of pain score			
Week 4	−1.9 ± 1.5 [−1.4 ± 1.6]	−1.6 ± 1.5 [−1.2 ± 1.5]	0.285 [0.786]∗
Week 12	−2.2 ± 1.1 [−1.5 ± 1.4]	−1.8 ± 1.4 [−1.4 ± 1.5]	0.151 [0.544]∗
Change of CGI-S			
Week 4	−3.1 ± 1.3 [−2.3 ± 1.7]	−3.0 ± 1.7 [−2.4 ± 1.9]	0.723 [0.630]∗
Week 12	−3.1 ± 1.7 [−2.2 ± 1.9]	−3.1 ± 1.5 [−2.5 ± 1.8]	0.870 [0.336]∗
CGI-I score at week 4	2.0 ± 1.2 [2.6 ± 1.3]	2.5 ± 1.4 [2.8 ± 1.4]	0.057 [0.368]∗
CGI-I score at week 12	2.0 ± 1.4 [2.6 ± 1.4]	2.4 ± 1.4 [2.7 ± 1.4]	0.183 [0.634]∗
Response rate at week 4	24 (54.5) [24 (40.6)]	17 (39.5) [30.3]	0.117 [0.469]∗∗
Response rate at week 12	32 (72.7) [32 (54.2)]	23 (53.4) [23 (41.0)]	0.0503 [0.416]∗∗

Data are presented as mean ± SD. Data of the intention to treat analysis are shown in []. CGI-S, CGI-I: Clinical Global Impression Severity and Improvement Scales. ∗Mann-Whitney *U* test; ∗∗Chi-square test.

**Table 3:** Differences of side effect in mebeverine and placebo groups.

	Mebeverine *n* = 44	Placebo *n* = 43	*P* value
Insomnia	4 (9.0)	1 (2.3)	0.195
Nausea	3 (6.8)	1 (2.3)	0.317
Drowsiness	8 (18.1)	7 (16.2)	0.540
Dry mouth	19 (43.1)	10 (23.2)	0.047
Diarrhea	0	0	—
Vomiting	1 (2.2)	0	0.512
Fatigue	4 (9.0)	6 (13.9)	0.340
Headache	3 (6.8)	1 (2.3)	0.326
Dizziness	2 (4.5)	2 (4.6)	0.674
Allergic reaction	0	0	—
Loss of appetite	8 (18.1)	8 (18.6)	0.568

Data are presented as number (%).

## References

[1] American Academy of Pediatrics Subcommittee on Chronic Abdominal Pain (2005). Chronic abdominal pain in children. *Pediatrics*.

[2] Rasquin A, Di Lorenzo C, Forbes D (2006). Childhood functional gastrointestinal disorders: child/adolescent. *Gastroenterology*.

[3] Chitkara DK, Rawat DJ, Talley NJ (2005). The epidemiology of childhood recurrent abdominal pain in western countries: a systematic review. *American Journal of Gastroenterology*.

[4] Saps M, Seshadri R, Sztainberg M, Schaffer G, Marshall BM, di Lorenzo C (2009). A prospective school-based study of abdominal pain and other common somatic complaints in children. *Journal of Pediatrics*.

[5] Garber J, Zeman J, Walker LS (1990). Recurrent abdominal pain in children: psychiatric diagnoses and parental psychopathology. *Journal of the American Academy of Child and Adolescent Psychiatry*.

[6] Palermo TM (2000). Impact of recurrent and chronic pain on child and family daily functioning: a critical review of the literature. *Journal of Developmental & Behavioral Pediatrics*.

[7] Kellow JE, Delvaux M, Azpiroz F, Camilleri M, Quigley EMM, Thompson DG (1999). Principles of applied neurogastroenterology: physiology/motility-sensation. *Gut*.

[8] van Ginkel R, Voskuijl WP, Benninga MA, Taminiau JAJM, Boeckxstaens GE (2001). Alterations in rectal sensitivity and motility in childhood irritable bowel syndrome. *Gastroenterology*.

[9] Levy RL, Olden KW, Naliboff BD (2006). Psychosocial aspects of the functional gastrointestinal disorders. *Gastroenterology*.

[10] Di Lorenzo C, Colletti RB, Lehmann HP (2005). Chronic abdominal pain in children: a technical report of the American Academy of Pediatrics and the North American Society for Pediatric Gastroenterology, Hepatology and Nutrition. *Journal of Pediatric Gastroenterology and Nutrition*.

[11] Lemann M, Dederding JP, Flourie B, Franchisseur C, Rambaud JC, Jian R (1991). Abnormal perception of visceral pain in response to gastric distension in chronic idiopathic dyspepsia. The irritable stomach syndrome. *Digestive Diseases and Sciences*.

[12] Martínez-Vázquez MA, Vázquez-Elizondo G, González-González JA, Gutiérrez-Udave R, Maldonado-Garza HJ, Bosques-Padilla FJ (2012). Effect of antispasmodic agents, alone or in combination, in the treatment of Irritable Bowel Syndrome: systematic review and meta-analysis. *Revista de Gastroenterologia de Mexico*.

[13] Poynard T, Regimbeau C, Benhamou Y (2001). Meta-analysis of smooth muscle relaxants in the treatment of irritable bowel syndrome. *Alimentary Pharmacology and Therapeutics*.

[14] Ford AC, Talley NJ, Spiegel BMR (2008). Effect of fibre, antispasmodics, and peppermint oil in the treatment of irritable bowel syndrome: systematic review and meta-analysis. *British Medical Journal*.

[15] Darvish-Damavandi M, Nikfar S, Abdollahi M (2010). A systematic review of efficacy and tolerability of mebeverine in irritable bowel syndrome. *World Journal of Gastroenterology*.

[16] Kline RM, Kline JJ, di Palma J, Barbero GJ (2001). Enteric-coated, pH-dependent peppermint oil capsules for the treatment of irritable bowel syndrome in children. *Journal of Pediatrics*.

[17] Stinson JN, Kavanagh T, Yamada J, Gill N, Stevens B (2006). Systematic review of the psychometric properties, interpretability and feasibility of self-report pain intensity measures for use in clinical trials in children and adolescents. *Pain*.

[18] National Institute of Mental Health (1985). Rating scales and assessment instruments for use in pediatric psychopharmacology research. *Psychopharmacology Bulletin*.

[19] Khanna R, Macdonald JK, Levesque BG (2013). Peppermint Oil for the Treatment of Irritable Bowel Syndrome: a systematic review and meta-analysis. *Journal of Clinical Gastroenterology*.

[20] Hills JM, Aaronson PI (1991). The mechanism of action of peppermint oil on gastrointestinal smooth muscle: an analysis using patch clamp electrophysiology and isolated tissue pharmacology in rabbit and guinea pig. *Gastroenterology*.

[21] McKay DL, Blumberg JB (2006). A review of the bioactivity and potential health benefits of peppermint tea (Mentha piperita L.). *Phytotherapy Research*.

[22] Irvine EJ, Whitehead WE, Chey WD (2006). Design of treatment trials for functional gastrointestinal disorders. *Gastroenterology*.

